# Bilingual Exposure and Sex Shape Developmental Trajectories of Brain Responses to Speech-Sound Features in Infants

**DOI:** 10.1162/NOL.a.214

**Published:** 2026-01-13

**Authors:** Marta Puertollano, Natàlia Gorina-Careta, Siham Ijjou-Kadiri, Alejandro Mondéjar-Segovia, María Dolores Gómez-Roig, Carles Escera

**Affiliations:** Brainlab – Cognitive Neuroscience Research Group, Department of Clinical Psychology and Psychobiology, University of Barcelona, Barcelona, Catalonia, Spain; Institute of Neurosciences, University of Barcelona, Barcelona, Catalonia, Spain; Institut de Recerca Sant Joan de Déu, Esplugues de Llobregat, Barcelona, Catalonia, Spain; BCNatal – Barcelona Center for Maternal Fetal and Neonatal Medicine, Hospital Sant Joan de Déu and Hospital Clínic, University of Barcelona, Barcelona, Catalonia, Spain; Primary Care Interventions to Prevent Maternal and Child Chronic Diseases of Perinatal and Developmental Origin Network (RICORS), Instituto de Salud Carlos III, Madrid, Spain

**Keywords:** auditory evoked potential, bilingualism, frequency-following response (FFR), infants, sex, speech encoding

## Abstract

As the auditory brain becomes functional during the third trimester of pregnancy, both biological and environmental processes begin shaping its maturation, influencing how speech sounds are perceived. Biological factors, such as sex, introduce early genetic differences, while environmental experiences, like bilingualism, modulate the auditory input that infants receive. Although existing research highlights the impact of sex and bilingualism on the development of speech perception, the neural mechanisms remain unclear. In this study, we recorded frequency-following responses longitudinally, at birth, 6 months, and 12 months of age in 73 infants exposed to varying degrees of bilingual input. We modeled the developmental trajectories for neural encoding of voice pitch and speech formant structure, finding significant maturation during the first 6 months, followed by less pronounced change through the first year. Distinct developmental patterns emerged as a function of sex and bilingualism, revealing their influence on neural attunement to key speech-sound features. Female infants exhibited stronger neural encoding of both pitch and formant structure, depicting a distinctive quadratic trajectory that peaked at 6 months. Bilingual exposure notably predicted lower neural pitch encoding values at 6 months, but higher values by 12 months. A positive effect of bilingualism on speech formant encoding was observed throughout the first year. These findings reveal how biological and environmental factors contribute to individual variability in early auditory development and speech acquisition.

## INTRODUCTION

Speech acquisition initiates at a very early developmental stage, as the auditory brain is already functional and able to process sounds since the beginning of the third trimester of pregnancy (Hepper & Shahidullah, 1994; Moore & Linthicum, 2007; Querleu et al., 1988; Ruben, 1995). It is around the 27th week of gestation that hearing becomes fully functional and the first fetal responses to sounds can be registered (Draganova et al., 2018; Schneider et al., 2001). At this fetal stage, the cochlea and the temporal lobe are formed and myelination appears through the brainstem and up to the auditory thalamus (Lavigne-Rebillard & Bagger-Sjöbäck, 1992; Moore & Linthicum, 2007; Moore et al., 1995). Although the exact acoustic features reaching the fetus remain unclear, intrauterine recordings suggest that the acoustic signal is altered by the maternal womb, which attenuates around 30 dB frequencies above 500 Hz (Abrams et al., 2000; Gerhardt & Abrams, 1996, 2000). This filtering primarily preserves the prosodic features of speech, conveying the variations in pitch, loudness, and rhythm, while suppressing the phonemic contrast information (Moon, 2017; Querleu et al., 1988).

It is during this early period that fetuses are first exposed to an acoustic environment, which significantly influences the development of their acoustic capacities (Arenillas-Alcón et al., 2023; Gorina-Careta et al., 2024; Moon et al., 2013; Partanen et al., 2013). For instance, daily music exposure during pregnancy has been shown to positively impact the neural encoding of speech sounds at birth (Arenillas-Alcón et al., 2023). Similarly, prenatal exposure to a bilingual environment affects the neonatal acoustic sensitivity to speech frequencies (Gorina-Careta et al., 2024). Neonates also demonstrate distinct preferences that indicate prenatal acoustic learning and tuning to the prosody of their native language (DeCasper & Fifer, 1980; Fernald & Simon, 1984; Granier-Deferre et al., 2011; Moon et al., 1993, 2013). This evidence suggests that such early exposure to speech-related acoustics may lay the foundation for postnatal auditory development, potentially modulating how infants encode speech throughout their first year of life. Indeed, prenatal exposure to speech evokes enduring changes in neural dynamics that further support learning and memory (Mariani et al., 2023).

Shortly after birth, infants exhibit sensitivity to a wide range of linguistically significant distinctions (Gervain & Mehler, 2010; Martinez-Alvarez et al., 2023). Newborns are able to encode the pitch of speech sounds in an adult-like manner (Arenillas-Alcón et al., 2021) and discriminate between languages they have not been exposed to, provided those languages differ rhythmically (Byers-Heinlein et al., 2010; Mehler et al., 1988; Nazzi et al., 1998). However, language acquisition relies on the capacity to classify similar yet nonidentical sounds into either different or equivalent phonetic categories according to the specific language, which is dependent on further postnatal linguistic exposure (Kuhl et al., 1992, 2003; Rivera-Gaxiola et al., 2005). By the age of 6 months, infants typically begin to perceive the variability inherent in each phonetic unit, which enables them to identify vowels typical of their mother tongue and alters their phonetic perception toward a native-like model (Kuhl et al., 1992; Maye et al., 2002). These developmental changes are reflected in a pronounced enhancement in neural encoding of speech sound features (Puertollano et al., 2024; Ribas-Prats, Cordero, et al., 2023) and coincide with their first articulation of consonant-vowel sounds, marking the babbling stage (Oller, 1992).

Exposure to distinct linguistic environments further shape developmental trajectories for infant speech processing. Monolingual infants demonstrate precise discrimination among a wide range of native and non-native phonemic contrasts by 7 months of age (Best & McRoberts, 2003; Cheour et al., 1998; Rivera-Gaxiola et al., 2005). Conversely, bilingual infants at the same age do not exhibit equivalent levels of native phoneme discrimination, with within-group variability depending on the amount of exposure to each language (Best et al., 2016; García-Sierra et al., 2011). As infants approach their first year of life, their speech perception undergoes an experience-driven perceptual narrowing, leading to a refined attunement to their native phoneme repertoire (Cheour et al., 1998; Kuhl et al., 2006; Werker et al., 1981) and typically aligning with their first word productions (Fenson et al., 1994). As their expertise in native phoneme contrasts develops, the ability to discriminate non-native phonemes diminishes, becoming minimal by the end of the first year (Rivera-Gaxiola et al., 2005; Tsao et al., 2006; Werker & Tees, 1984). Notably, the timing of these developmental milestones is influenced by the balance of language input, regardless of whether infants are raised in monolingual or bilingual settings (García-Sierra et al., 2011, 2016).

Alongside linguistic environments, sex is a significant biological factor influencing speech processing. Beginning in the second trimester of pregnancy, the extensive placental transmission of sex-steroid hormones shapes early speech development (Lust et al., 2010; Lutchmaya et al., 2001; Schaadt et al., 2015; Wermke et al., 2014). As early as 1 month after birth, female infants tend to outperform males in phonological discrimination (Friederici et al., 2008), a difference mediated by sex hormone levels that are also linked to articulatory skills at 5 months (i.e., babbling; Quast et al., 2016) and to later language abilities in childhood (Hollier et al., 2013; Schaadt et al., 2015). Additionally, vocabulary growth rates have been observed to be faster in female infants (Dailey & Bergelson, 2023), while male infants face a higher risk of experiencing language delays within the first 3 years of life (Whitehouse et al., 2012). Despite these findings, there is still limited research exploring how sex modulates the developmental trajectories of neural speech encoding during the first year of life. Understanding such sex differences in speech development is crucial for addressing individual variability and establishing a clearer, sex-dependent normative framework for speech development.

Advancements in utilizing infant brain potentials have significantly pushed the study of neural mechanisms involved in speech perception and acquisition (Hervé et al., 2022). While mismatch negativity paradigms are well suited for assessing neural discrimination of auditory contrasts (Kujala et al., 2023), the frequency-following response (FFR) stands out as an auditory evoked potential that captures neural synchronization along the auditory pathway in response to complex sounds such as speech and music (Coffey et al., 2019; Gorina-Careta et al., 2021). FFR recordings have proven to be a powerful tool for investigating the neural encoding of speech-sound features, such as pitch and fine spectrotemporal details that underlie phoneme perception (Gorina-Careta et al., 2022; Krizman & Kraus, 2019). In infancy, the FFR has been employed to characterize typical and atypical development of neural speech encoding (Banai et al., 2005, 2009; Cunningham et al., 2001; Puertollano et al., 2024; Ribas-Prats et al., 2019, 2022; Ribas-Prats, Arenillas-Alcón, et al., 2023; Ribas-Prats, Cordero, et al., 2023). It has also been used to study biological and environmental influences on auditory processing, including sex-related differences throughout development (Krizman et al., 2019, 2020), as well as the impact of prenatal bilingual experiences in neonates (Gorina-Careta et al., 2024) and postnatal experiences in children (Krizman et al., 2015) and adults (Skoe et al., 2017).

Although individual studies have explored the effects of either sex or bilingualism on auditory encoding, no prior work has examined their individual effects on neural encoding of speech as well as their potential interaction during the first year of development. The present study aimed to uncover how age, perinatal bilingual experience (referred in this study as the period from the third trimester of gestation up to 1 postnatal year; Austin, 2014; Chiera et al., 2020), and sex collectively shape neural speech-encoding mechanisms during the first year of life. To this end, we recorded FFRs from infants with varying degrees of bilingual exposure and examined their developmental trajectories regarding the neural encoding of voice pitch and formant structure content. Building on previous research depicting neural encoding achievements during the first 6 months of life, which stabilize by the first year (Puertollano et al., 2024), we anticipated observing similar age-related effects. Additionally, we sought to find FFR neural correlates underlying the distinct developmental trajectories in phoneme discrimination associated with monolingual and bilingual experiences during infancy, as reported in previous behavioral and neurophysiological research (i.e., García-Sierra et al., 2011, 2016; Kuhl et al., 2006; Rivera-Gaxiola et al., 2005). We also expected to find sex-related differences in infant neural encoding of speech sounds, consistent with previous studies that demonstrate a female advantage on speech processing and speech related abilities at early developmental stages (i.e., Dailey & Bergelson, 2023; Friederici et al., 2008).

## MATERIALS AND METHODS

### Participants

A total of 159 healthy-term neonates were initially recruited at the Sant Joan de Déu (SJD) Barcelona Children’s Hospital (Catalonia, Spain) and tested at birth. Of these, 129 infants returned for a second FFR assessment at 6 months of age, and 97 participated in a third session at 12 months. From this longitudinal cohort, full linguistic background information was available for 77 infants who completed all three sessions. Four of these participants were classified as trilingual and were excluded from the final sample due to the small size of this subgroup, which could introduce confounding variability. The resulting final sample comprised 73 infants (38 females) with complete FFR and linguistic data across all three time points. Demographic characteristics of the final sample were as follows: mean gestational age at birth = 39.73 ± 0.97 weeks; mean birth weight = 3288.8 ± 302.6 grams; mean age at the neonatal session = 1.62 ± 0.94 days after birth; age at the 6-month session ranged from 5.36 to 7.40 months after birth (mean = 6.23 ± 0.37 months); and age at the 12-month session ranged from 11.81 to 13.22 months after birth (mean = 12.39 ± 0.36 months).

All infants participating in this study were born at term after low-risk gestations, with an adequate birth weight for their gestational age (Figueras & Gratacós, 2014). Any diagnosed pathology or an Apgar score below 7 at 1 and 5 minutes after birth were considered as exclusion criteria. None of the infants presented any risk factors for hearing impairment, as per the Joint Committee on Infant Hearing (2019) guidelines. As part of the standard medical routine to ensure auditory pathway integrity at birth, all neonates had passed the universal hearing screening test based on an Automated Auditory Brainstem Response system (ALGO 3i, Natus Medical Incorporated, San Carlos, CA).

Approval from the Bioethics Committee of SJD Barcelona Children’s Hospital (Internal Review Board ID: PIC-185-19) was obtained for this study. Prior to the infant data collection, all parents or legal guardians signed an informed consent in accordance with the Code of Ethics of the World Medical Association (Declaration of Helsinki).

### Language Exposure Measurement

Infants’ prenatal linguistic exposure was evaluated through a retrospective questionnaire delivered to their mothers (Gorina-Careta et al., 2024). Prenatal acoustic environment was considered as monolingual (i.e., no months of exposure) when mothers reported speaking only one language during their last trimester of pregnancy, or as bilingual (i.e., 3 months of exposure) when they reported using two different languages during that period. Postnatal linguistic exposure was evaluated for their first year of life through the Language Exposure Assessment Tool (LEAT; DeAnda et al., 2016), which provided the total number of months of bilingual exposure. Prenatal and postnatal exposure were analyzed within a unique continuous variable counting the number of months of bilingual exposure along the studied perinatal period (i.e., from the third trimester of pregnancy up to 1 year post-birth; Austin, 2014; Chiera et al., 2020). This approach was chosen over categorical classifications (e.g., low, medium, high exposure), which would have oversimplified the variability in the sample and failed to capture dynamic shifts in exposure across developmental stages. By treating bilingual exposure as a continuous variable that co-varies with age, we were able to account for developmental fluctuations in their language environment.

To ensure that bilingual exposure was comparable between female and male infants across development, we conducted Wilcoxon rank-sum tests comparing bilingual exposure values between female and male infants at each of the three measured time points (birth, 6 months, and 12 months). These analyses confirmed no significant differences at any age (Bonferroni-corrected *p* values: birth, *W* = 569.5, *p* = 0.65; 6 months, *W* = 620.0, *p* = 1; 12 months, *W* = 579.5, *p* = 1).

Bilingual exposure in the sample was characterized by the combination of Spanish and other language, being for most of the cases the Spanish-Catalan combination (89.3%). Other languages heard by infants were Bulgarian, English, Galego, Guarani, Moroccan Arabic, and Portuguese (see more details in Table 1). Languages composing monolingual environments in the sample were either Spanish (88.2%) or Catalan (11.8%).

**Table 1.** Languages heard by infants from the third trimester of pregnancy to 12 months of age

Languages	*N*	%
Spanish	15	20.5
Catalan	2	2.7
Spanish–Catalan	49	67.1
Spanish–Other	7	9.7
Bulgarian	1	1.4
English	1	1.4
Galego	1	1.4
Guarani	2	2.7
Moroccan Arabic	1	1.4
Portuguese	1	1.4

### Stimulus

**Figure 1.**
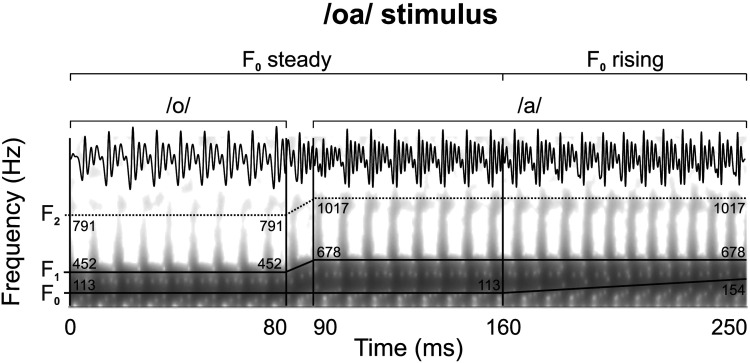
Temporal and spectral representation of the /oa/ stimulus with a schematic illustration of its formant structure. The figure pictures the stimulus’ frequency decomposition associated to the different stimulus sections: F_0_ (113 Hz), F_1_ (452 Hz during the /o/ section, 678 Hz during the /a/ section) and F_2_ (791 Hz during the /o/ section, 1017 Hz during the /a/ section).

Neural responses were obtained to a 250 ms two-vowel /oa/ speech stimulus with a rising pitch ending (see Figure 1; for a detailed description, see Arenillas-Alcón et al., 2021). The /oa/ stimulus was used for the study as it allows the assessment of the infant neural responses to two vowels with distinct phonetic contrasts. Three different sections can be differentiated in the stimulus according to its F_0_ and F_1_: the /o/ section (from 10 to 80 ms; F_0_ = 113 Hz; F_1_ = 452 Hz), the /a/ steady section (from 90 to 160 ms; F_0_ = 113 Hz; F_1_ = 678 Hz) and the /a/ rising section (from 160 to 250 ms; F_0_ = 113–154 Hz; F_1_ = 678 Hz). For the analysis of F_0_ encoding, the two pitch-steady sections of the stimulus (i.e., the /o/ and the /a/ steady sections) were combined into a single analysis window, referred to as the stimulus steady section (from 10 to 160 ms; F_0_ = 113 Hz). The inclusion of a rising pitch trajectory enables a more detailed assessment of infant neural encoding of pitch dynamics encountered in speech and their modulation by sex and bilingual exposure.

The specific vowels’ F_1_ were chosen as those belong to the prototypical phonetic repertoire in both Spanish and Catalan languages (Alarcos Llorach, 1965; Martí i Roca, 1986). Following the guidelines of previous neonatal and infant FFR research (Arenillas-Alcón et al., 2021; Gorina-Careta et al., 2022; Ribas-Prats et al., 2019), as well as to provide reliable comparisons with prior findings in the field (i.e., Arenillas-Alcón et al., 2023; Puertollano et al., 2024; Ribas-Prats et al., 2019; Ribas-Prats, Cordero, et al., 2023), the stimulus was presented at a rate of 3.39 Hz and an intensity of 60 dB SPL, monaurally to the right ear. It was delivered in alternating polarities through an earphone connected to a Flexicoupler disposable adaptor (Natus Medical Incorporated, San Carlos, CA).

### Procedure and Data Acquisition

Neonatal FFR responses were recorded while the babies were sleeping in their crib at the hospital room. FFR sessions at 6 and 12 months of age were conducted at a hospital dispensary while ensuring the infant remained either asleep or as calm as possible, aiming to guarantee the highest quality of data. The recording sessions had a total mean duration of around 30 minutes, including 5 minutes of preparation time, 20 minutes of recording (4 /oa/ blocks × 1,000 sweeps × 295 ms stimulus-onset asynchrony), and up to 5 minutes of additional time for the rejected sweeps.

The speech stimulus was presented using a SmartEP platform connected to a Duet amplifier, which includes the cABR and Advanced Hearing Research modules (Intelligent Hearing Systems, Miami, FL, USA). Neural responses were recorded using three disposable Ag/AgCl electrodes placed in a vertical montage (active electrode located at Fpz, ground at the forehead and reference at the right mastoid), keeping impedances below 10 kΩ for all electrodes. The continuous FFR signal acquisition was completed at a sampling rate of 13333 Hz utilizing an online band-pass filter to eliminate frequencies outside the 30 to 1500 Hz range. Electroencephalography online data was epoched from −40.95 (prestimulus period) to 249.975 ms, automatically excluding any sweep with voltage values exceeding ±30 *μ*V.

### Data Processing

Acquired FFRs were band-pass filtered from 80 to 1500 Hz. To emphasize the FFR components associated to the speech stimulus envelope (FFR_ENV_) and to diminish putative cochlear microphonics, neural responses to alternating polarities were averaged [(Condensation + Rarefaction)/2]. To assess the neural encoding of the stimulus’ F_1_, and minimizing the envelope related neural activity (Aiken & Picton, 2008; Krizman & Kraus, 2019), the FFR temporal fine structure (FFR_TFS_) was obtained by subtracting neural responses to the two opposite polarities [(Rarefaction − Condensation)/2].

FFR parameters were estimated using custom scripts from MATLAB R2019b (MathWorks, 2019) used in previous similar studies (Arenillas-Alcón et al., 2021; Ribas-Prats et al., 2019). A detailed description can be found below for the three FFR parameters separately extracted and tested for the different stimulus features of interest.

#### Spectral amplitude

Spectral amplitude (in nV) was obtained as an indicator of the neural-phase locking magnitude at the frequency of interest (F_0_, 113 Hz; /o/ F_1_, 452 Hz; /a/ F_1_, 678 Hz) (Arenillas-Alcón et al., 2021; Ribas-Prats et al., 2019; White-Schwoch et al., 2015). It was calculated by applying the fast Fourier transform (Cooley & Tukey, 1965) to the neural response. Spectral amplitude was defined as the mean amplitude within a ±5 Hz window centered at the frequency peak of interest. Spectral amplitude at F_0_ was obtained from the FFR_ENV_ corresponding to the stimulus steady section (10 to 160 ms) to quantify voice-pitch encoding of the speech-sound stimulus. Spectral amplitudes at the vowels’ F_1_ frequencies were retrieved separately from the FFR_TFS_ corresponding to the /o/ section (10 to 80 ms) and the /a/ steady section (90 to 160 ms).

#### Pitch error

Pitch error (in Hz) was extracted from the FFR_ENV_ as a measure of pitch encoding accuracy for the F_0_ contour along the two /a/ sections of the stimulus (i.e., /a/ steady section and /a/ rising section). It was computed as an average of the absolute Euclidian distance between the stimulus and response F_0_ from 40-ms time bins separately for the two sections mentioned.

### Statistical Analysis

Statistical analyses were performed using Jamovi (Version 2.4.11; Jamovi Project, 2024). To explore the effects of perinatal bilingual exposure and sex on the developmental trajectory of neural encoding of speech, linear mixed effects models were constructed separately for each FFR parameter according to our hypothesis: spectral amplitude (at 113 Hz, 452 Hz and 678 Hz) and pitch error (during the /a/ steady and /a/ rising). Normality was assessed with Kolmogorov-Smirnov test and a natural logarithm (ln) transformation was applied to spectral amplitude dependent variables, as those did not meet such assumption. Five different models were created to explore the trajectory of neural encoding of speech-sound characteristics, one per each dependent variable, as follows:

$$\text{FFR}\;\text{parameter}∼\text{age}+\text{sex}+\text{bilingual}\;\text{exposure}+\text{age}*\text{sex}+\text{age}*\text{bilingual}\;\text{exposure}+\left(1|\text{subject}\right)$$

Individual tested models predicted each FFR value as a function of age (birth, 6 and 12 months) and sex (male, female) and bilingual perinatal exposure (as a covariate, ranging from 0 to 15 months of exposure). The models also included the interaction effect of age per sex, and age per bilingual exposure. A by-subject random intercept was included in the models to account for infants’ repeated measures.

A trend analysis was performed within each separated model by coding the age variable with polynomial contrasts, which describe possible trends in the means (i.e., shape of the age-dependent trend). We used polynomial contrasts to enable modeling of possible nonlinear developmental changes in neural encoding of speech during the first year of life (see Puertollano et al., 2024). The polynomial regression was thus applied to depict the relationship between the FFR parameters and age to find the best way to draw a line through the data points. After a significant result for the omnibus test of fixed effects, post hoc analysis of variance or multiple comparison with Bonferroni adjustments were conducted to further inspect the given result. Only Bonferroni-corrected *p* values are reported in the Results section. Normality of residuals was met for each independent model, which was checked using the Kolmogorov-Smirnov test.

## RESULTS

**Figure 2.**
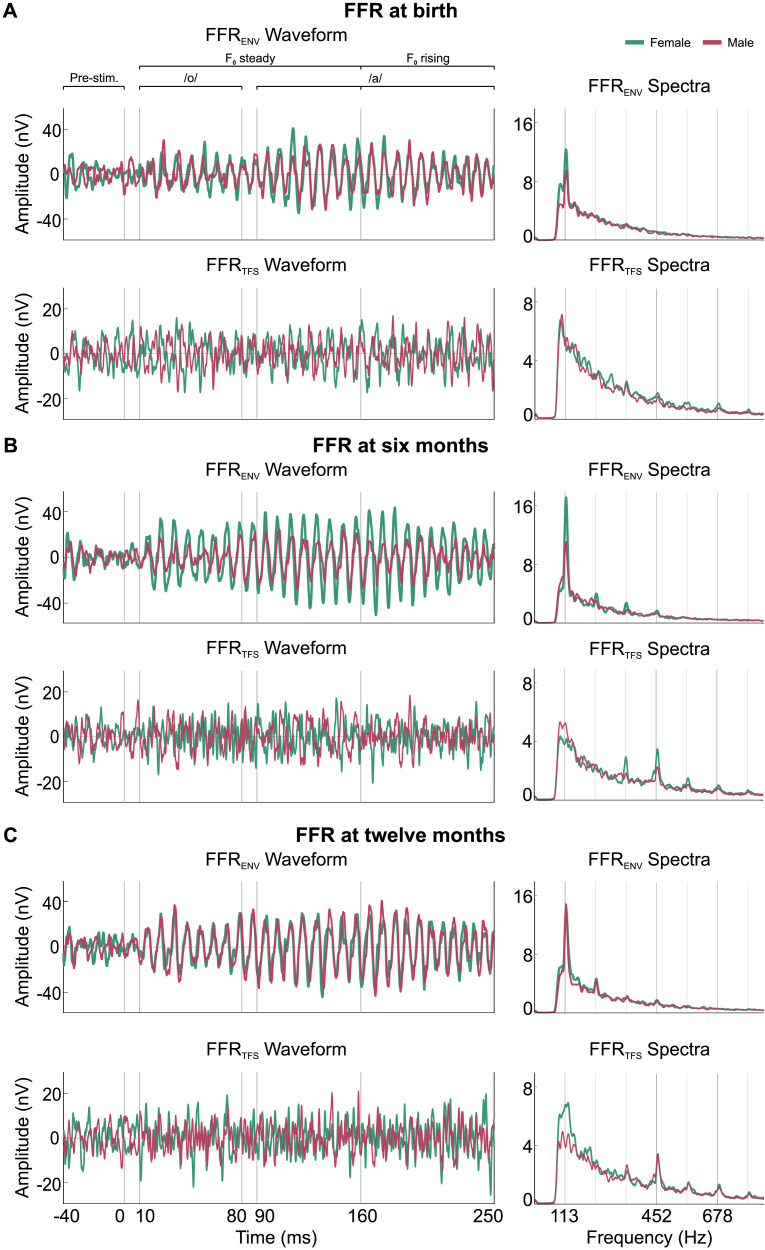
Frequency-following responses (FFRs) from 38 female (in green) and 35 male (in red) infants recorded at each developmental stage: (A) at birth, (B) at 6 months, and (C) at 12 months. The left column displays grand-averaged speech stimulus envelope (FFR_ENV_) and temporal fine structure (FFR_TFS_) waveforms in the time domain for each age group. The right column illustrates the amplitude spectra of FFR_ENV_ and FFR_TFS_, both extracted from the steady section of the stimulus (10–160 ms). Remarkably, a clear sex difference in F_0_ encoding emerges at 6 months of age, with females displaying larger amplitudes than males.

Clear FFRs were elicited across each developmental stage (i.e., at birth, 6 months and 12 months) in both female and male infants. These neural responses are illustrated in Figure 2, as FFR waveforms and corresponding spectral representations. As can be observed in the figure, spectral peaks are present at F_0_ across ages, and for the stimulus F_1_ they emerge from 6 months onwards.

### Voice Pitch Encoding

#### Spectral amplitude at stimulus F_0_

**Figure 3.**
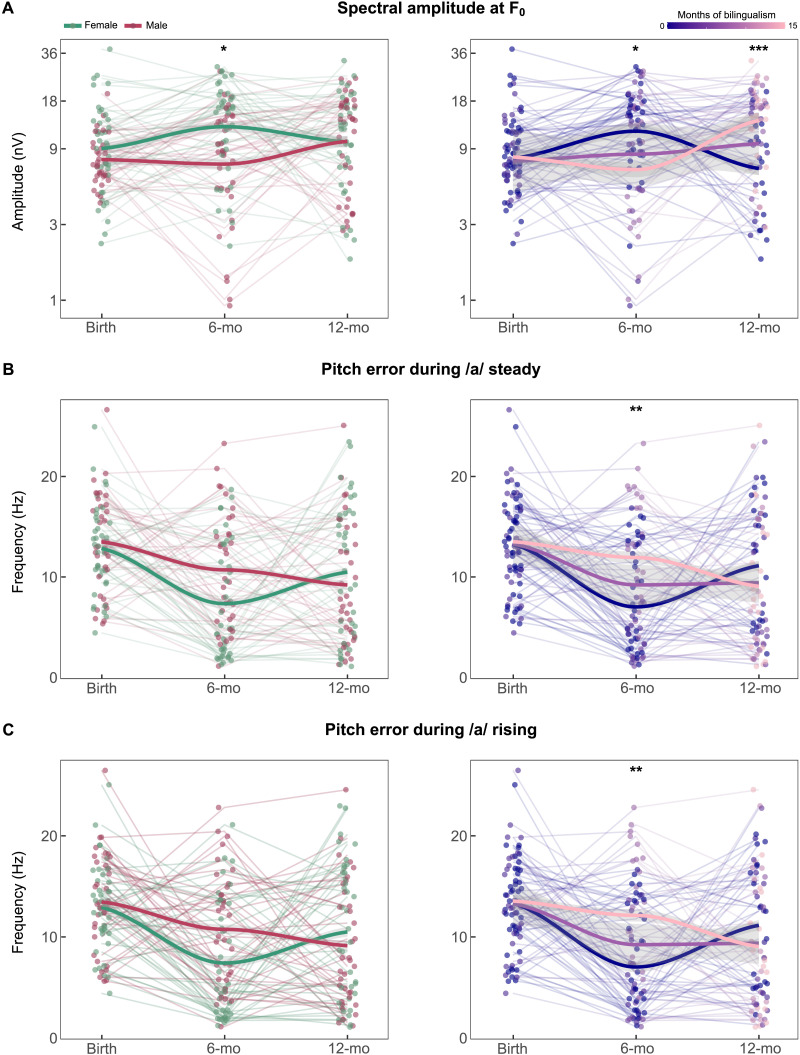
Predicted developmental trajectories and pitch error during sections of the stimulus across the three developmental stages: at birth, 6 months and 12 months. (A) Spectral amplitudes at F_0_. (B) Pitch error during /a/ steady section. (C) Pitch error during rising section. Solid lines illustrate the predicted trajectories based on sex (left panels: female in green, male in red) and bilingual exposure (right panels: no exposure in dark blue, median exposure in purple, maximum exposure in pink). Gray shaded regions illustrate confidence intervals for the predicted trajectories. Post hoc significant results within developmental stages are labeled as follows: *p < 0.05, **p < 0.01, ***p < 0.001. Each data point in A represents the log-transformed spectral amplitudes of the longitudinally tested infants to account for skewness. For interpretability, the *y*-axis displays the corresponding real spectral amplitude values. Data points displayed in B and C represent the nontransformed pitch error values corresponding to each tested infant.

The model fit was statistically significant (*X*^2^(9) = 37.544; R^2^ = 0.275; *p* < 0.001). The regression results for the model indicated a main effect of sex (*β* = −0.242; *t*(70.6) = −2.312; *p* = 0.024), with general higher spectral amplitudes shown by female infants (*M* = 0.013 ± 0.008) in comparison to their male peers (*M* = 0.01 ± 0.006; see Figure 3A). The interaction effect between age and sex was found to be a significant predictor of the spectral amplitude at 113 Hz (*F*_(2,140.3)_ = 3.15; *p* = 0.046). Specifically, polynomial contrasts revealed a significant interaction between the quadratic effects of age and sex (*β* = 0.347; *t*(140.2) = 2.43; *p* = 0.016). Simple slopes analysis revealed that the quadratic trajectory of spectral amplitude as a function of age was only present for female infants (*β* = −0.237; *t*(156) = −2.01; *p* = 0.047). Post hoc analyses showed higher values for female in comparison to male infants at the age of 6 months (*t*(199) = 3.36; *p* = 0.014). Other pairwise comparisons did not reach statistical significance after Bonferroni corrections (*p* > 0.05).

The interaction effect between age and bilingual exposure was also significant (*F*_(2,173.6)_ = 8.19; *p* < 0.001; see Figure 3A). Specifically, the quadratic effect of age per bilingual exposure interaction was found as a significant predictor (*β* = 0.062; *t*(161.1) = 2.33; *p* = 0.021), with simple slopes analysis revealing that only for monolingual infants (i.e., none-exposed to bilingualism) a significant quadratic effect of age was predicted on spectral amplitude at 113 Hz (*β* = −0.340; *t*(142.1) = −3.06; *p* = 0.003). Simple slopes analysis further revealed a negative effect of bilingual exposure on spectral amplitudes at 6 months (*β* = −0.051; *t*(209.4) = −2.42; *p* = 0.017), along with a positive effect at 12 months (*β* = 0.043; *t*(209.9) = 3.27; *p* = 0.001).

#### Pitch error during the /a/ steady section

The model fit was statistically significant (*X*^2^(9) = 38.618; R^2^ = 0.232; *p* < 0.001). The regression results for the model indicated a main effect of age (*F*_(2,176.9)_ = 3.58; *p* = 0.030), with higher values at birth (*M* = 13.25 ± 1.43) in comparison to 6 months of age (*M* = 9.19 ± .66; *t*(194.1) = 2.64; *p* = 0.027) and depicting a significant quadratic trajectory (*β* = 2.09; *t*(162.9) = 2.55; *p* = 0.012). The interaction between the quadratic effect of age and sex was found as a significant predictor of pitch error during the /a/ steady section (*β* = −2.826; *t*(140.5) = −2.25; *p* = 0.026; see Figure 3B), with simple slopes analysis revealing a quadratic trajectory only significantly present for female infants (*β* = 3.504; *t*(156.8) = 3.39; *p* < 0.001). Post hoc pairwise comparison showed lower pitch error values for females at 6 months (*M* = 7.58 ± 0.92) in comparison to male neonates (*M* = 13.57 ± 1.51; *t*(209.8) = 3.40; *p* = 0.012). Other comparisons did not reach statistical significance after Bonferroni adjustments.

The interaction effect between age and bilingual exposure was also found to be a significant predictor (*F*_(2,175.1)_ = 4.31; *p* = 0.015; see Figure 3B). Post hoc estimates revealed higher pitch error values as a function of bilingual exposure at 6 months of age (*β* = 0.470; *t*(209.7) = 2.62; *p* = 0.009). However, neither linear (*β* = −0.110; *t*(200) = −0.34; *p* = 0.73) nor quadratic contrasts (*β* = −0.421; *t*(164.1) = −1.83; *p* = 0.07) of the age per bilingual exposure interaction significantly predicted pitch error during the /a/ steady section.

#### Pitch error during the /a/ rising section

The model fit was statistically significant (*X*^2^(9) = 39.642; R^2^ = 0.241; *p* < 0.001). The regression results for the model indicated a main effect of age (*F*_(2,176.9)_ = 3.51; *p* = 0.032), with higher values at birth (*M* = 13.28 ± 1.42) in comparison to 6 months of age (*M* = 9.25 ± 0.66; *t*(194) = 2.62; *p* = 0.028) and depicting a quadratic effect of age as a significant predictor of pitch error during the /a/ rising section (*β* = 2.04; *t*(162.7) = 2.50; *p* = 0.013). The interaction between age and sex was found as a significant predictor (*F*_(2,140.6)_ = 3.14; *p* = 0.046). Moreover, the quadratic effects of age per sex interaction was also significant (*β* = −2.871; *t*(140.5) = −2.30; *p* = 0.023; see Figure 3C). Simple slopes analysis revealed that the quadratic trajectory of pitch error as a function of age was only present for female infants (*β* = 3.474; *t*(156.7) = 3.38; *p* < 0.001). Post hoc comparisons revealed higher values for female infants at 6 months in comparison to male neonates (*t*(209.8) = 3.35; *p* = 0.014). Other pairwise comparisons did not reach statistical significance (*p* > 0.05).

The interaction effect between age and bilingual exposure was also found to be a significant predictor (*F*_(2,175)_ = 4.72; *p* = 0.010; see Figure 3C). Post hoc estimates revealed higher pitch error values as a function of bilingual exposure at 6 months of age (*β* = 0.491; *t*(209.7) = 2.75; *p* = 0.006). However, neither linear (*β* = −0.111; *t*(199.6) = −0.34; *p* = 0.73) nor quadratic trajectories (*β* = −0.440; *t*(163.8) = −1.92; *p* = 0.06) of the age per bilingual exposure interaction were found as significant predictors in the model.

### Formant Structure Encoding

#### Spectral amplitude at /o/ vowel F_1_

**Figure 4.**
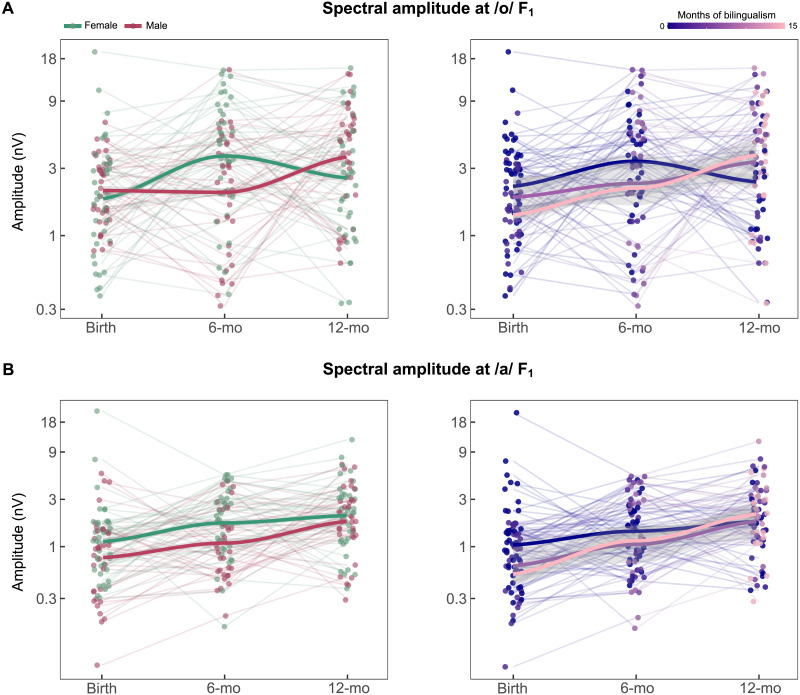
Predicted developmental trajectories of neural encoding across the three developmental stages: at birth, 6 months and 12 months. (A) /o/ F_1_. (B) /a/ F_1_. Each point represents the log-transformed amplitudes for each longitudinally tested infant to account for skewness. For interpretability, the *y*-axis displays the corresponding real spectral amplitude values. The solid lines represent the predicted trajectories according to sex (left panels: female in green, male in red) and bilingual exposure (right panels: no exposure in dark blue, median exposure in purple, maximum exposure in pink), with gray shaded regions illustrating confidence intervals.

Model fit was statistically significant (*X*^2^(9) = 30.512; R^2^ = 0.211; *p* < 0.001). The regression results for the model indicated a main effect of age (*F*_(2,176.6)_ = 4.77; *p* = 0.010), with lower values at birth (*M* = 0.003 ± 0.003) in comparison to both 6 months (*M* = 0.004 ± 0.004; *t*(193.8) = −2.93; *p* = 0.011) and 12 months of age (*M* = 0.004 ± 0.004; *t*(208.2) = −2.95; *p* = 0.010). Both a linear (*β* = 0.526; *t*(208.2) = 2.96; *p* = 0.003) and a quadratic (*β* = −0.276; *t*(162.2) = −2.15; *p* = 0.033) effect of age resulted as significant predictors of spectral amplitude at 452 Hz. The interaction between age and sex was found to a be significant predictor (*F*_(2,140.2)_ = 6.15; *p* = 0.003; see Figure 4A), as well as the quadratic trajectory of age by sex interaction (*β* = 0.684; *t*(140.0) = 3.48; *p* < 0.001). Post hoc results showed a significant quadratic effect of age only for female participants (*β* = −0.618; *t*(156) = −3.81; *p* < 0.001), with 6-months old females showing higher spectral amplitudes in comparison to both male (*t*(210) = −3.27; *p* = 0.019) and female neonates (*t*(184) = −3.83; *p* = 0.003). Significantly higher spectral amplitudes were also depicted for 12-months male infants in comparison to female neonates (*t*(204) = −3.23; *p* = 0.022).

The interaction between age and bilingual exposure was also significant (*F*_(2,174.6)_ = 3.41; *p* = 0.035; see Figure 4A), as it was the linear effect of age per bilingual exposure interaction (*β* = 0.115; *t*(199.3) = 2.26; *p* = 0.025). Simple slopes analysis revealed a predicted linear effect of age specifically for infants with medium level of bilingual exposure (7.5 months of exposure; *β* = 0.920; *t*(207.8) = 2.81; *p* = 0.005) and for those with maximum bilingual exposure (15 months of exposure; *β* = 1.780; *t*(204.7) = 2.56; *p* = 0.011), while not present for monolinguals (i.e., nonexposed to bilingualism; *p* > 0.05). Post hoc estimate results did not reach statistical significance at any specific developmental stage.

#### Spectral amplitude at /a/ vowel F_1_

Model fit was statistically significant (*X*^2^(9) = 47.423; R^2^ = 0.323; *p* < 0.001). The regression results indicated a main effect of age (*F*_(2,176.7)_ = 10.75; *p* < 0.001), further revealing a linear effect of age as a significant predictor of spectral amplitude at 678 Hz (*β* = 0.769; *t*(208.4) = 4.61; *p* < 0.001). Post hoc estimates depicted lower values at birth (*M* = 0.0015 ± 0.003) in comparison to both 6 months (*M* = 0.0018 ± 0.001; *t*(193.1) = −3.53; *p* = 0.002) and 12 months of age (*M* = 0.0025 ± 0.002; *t*(208.4) = −4.61; *p* < 0.001). A main effect of sex was also discovered (*β* = −0.309; *t*(70.9) = −2.28; *p* = 0.026; see Figure 4B), with generally higher values for female infants (*M* = 0.0022 ± 0.003) than their male peers (*M* = 0.0016 ± 0.001). Neither linear nor quadratic trajectories for the age by sex interaction were found as significant predictors.

The linear effect of age per bilingual exposure interaction was also found as a significant predictor (*β* = 0.111; *t*(196.2) = 2.36; *p* = 0.019; see Figure 4B), specifically indicating that the linear trajectory of age on spectral amplitude at 678 Hz depends on the level of bilingual exposure. Simple slopes analysis revealed a predicted positive linear effect of age for all three contrasted levels of bilingual exposure: none exposed infants (*β* = 0.317; *t*(159.6) = 2.40; *p* = 0.018), infants exposed during 7.5 months (*β* = 1.151; *t*(206.9) = 3.75; *p* < 0.001), and infants exposed during 15 months to bilingual exposure (*β* = 1.986; *t*(202.8) = 3.07; *p* = 0.002).

## DISCUSSION

The present study was set to investigate the distinct trajectories in the neural encoding of speech-sound features across the first year of infant development, with a focus on the influences of sex and perinatal bilingual exposure. To that aim, we inspected FFR neural responses to a two-vowel /oa/ syllable at birth, 6 months, and 12 months of age. We then modeled the neural encoding of both pitch and formant structure over time, analyzing variations according to bilingualism and sex. Our findings provide new insights into how sex and early bilingual exposure shape the neural encoding of speech sounds in infancy, with potential implications for understanding the individual variability in early language acquisition.

Our results reveal a significant maturation of neural encoding for both voice pitch and formant structure of speech during the first 6 months of life, followed by continued refinement over the second half of the year. Notably, different developmental trajectories were observed for each of the different speech features of interest. While the encoding of both steady and rising F_0_ contours followed a quadratic trajectory, the encoding of both vowels’ F_1_ exhibited a linear progression. Interestingly, the encoding of the /o/ F_1_ frequency fit both linear and quadratic models, suggesting a more complex developmental pattern. These divergent trajectories may reflect differences related to the development of low- and high-frequency acoustic information processing, paralleling the spectrally ascendant developmental pattern of the auditory system (Graven & Browne, 2008). Accordingly, neural attunement to low-frequency acoustic content initiates earlier, the period from 25 gestational weeks to 6 postnatal months being the most critical for the neurosensory development of the auditory system. This aligns with the earlier availability of low-frequency acoustic signals accessible to the fetus during the prenatal period (Hepper & Shahidullah, 1994; Voegtline et al., 2013) and is supported by previous research demonstrating robust pitch encoding already at birth (Arenillas-Alcón et al., 2021; Jeng et al., 2011). Intriguingly, although our findings did not reveal significant age-related changes in spectral amplitudes at F_0_, we did identify marked improvements in the neural tracking of both steady and rising pitch contours within the first 6 months, aligning with previous research depicting higher pitch tracking during the first year of life (Wong et al., 2021). These results may help clarify inconsistencies in previous research on infant age-related changes in pitch encoding, which may stem from subtle variations in the features examined (Anderson et al., 2015; Jeng et al., 2010; Puertollano et al., 2024; Ribas-Prats, Cordero, et al., 2023; Van Dyke et al., 2017).

On the other hand, the neural encoding of F_1_ components has been documented to experience significant enhancement already in the first postnatal month (Ribas-Prats, Cordero, et al., 2023), suggesting the rapid neural adaptation to novel frequencies that become significantly more available after birth. Our results corroborate previous research demonstrating further refinement of F_1_ neural encoding during the first 6 months of life (Ribas-Prats, Cordero, et al., 2023) along with a more gradual development up to the age of 12 months (Puertollano et al., 2024), but further extend these findings by revealing a linear maturational pattern throughout the first year of life. This underscores the early development of neural mechanisms crucial for establishing a native language’s sound map by 6 months, thereby facilitating native phoneme identification and discrimination (Kuhl, 1991, 2004, 2010; Kuhl et al., 1992).

A complementary explanation for this early maturation pattern lies in the constraints imposed by neuronal development, as shown by the evolution of electrophysiological brain activity in early infancy. Fetal and neonatal brain activity is primarily characterized by slow-wave oscillations that match the slow prosodic modulations found in speech. By around 6 months of age, faster neuronal oscillations that can phase-lock to phoneme-rate amplitude modulations emerge (Anderson & Perone, 2018; Le Van Quyen et al., 2006; Xu et al., 2011; for a review see Menn et al., 2023), enabling infants to encode higher frequency speech components and build a repertoire of native phonemes (Kuhl, 2004). Neural encoding of phonetic features becomes robust by 7 months of age and shows no further enhancement up to the age of 11 months (Di Liberto et al., 2023). Similarly, our findings highlight the first 6 months as a sensitive period, when the neural system increasingly attunes to the spectro-temporal features of the acoustic environment along the auditory pathway, setting the stage for subsequent phonetic learning (Werker & Hensch, 2015; Zhao et al., 2022).

There is a vast body of literature emphasizing the complex interplay between maturational and experiential influences on speech development (for a review see Werker & Hensch, 2015). Evidence for a sensitive period for phonetic attunement occurring during the first 12 months of life comes from diverse biological and environmental contexts. While studies on infants born prematurely emphasize the relevance of maturational processes in speech perception development (Peña et al., 2010, 2012), research into acoustic deprivation due to infant deafness underscores the critical role of sensory input in driving acoustic-related neural plasticity during the first year of life (Faulkner & Pisoni, 2013; Kral & Sharma, 2012). A particular acoustic environment is that related to bilingual experience, which has been previously proposed to modulate the onset and duration of the sensitive period for phonetic learning (Best et al., 2016; García-Sierra et al., 2011; Werker et al., 2009; Werker & Hensch, 2015). This extended sensitive period likely offers bilingual infants additional time to attune to the acoustic and phonological features of both languages. Our findings support this proposal by underscoring the moderating influence of bilingualism on the development of neural mechanisms involved in pitch and formant structure encoding, both of which are essential for speech and language acquisition.

We observed a negative impact of bilingual exposure on voice pitch encoding at 6 months, as reflected by lower spectral amplitudes at F_0_ and higher pitch error values. This trend reversed by 12 months, with bilingual exposure predicting higher spectral amplitudes at F_0_. This U-shaped pattern could reflect an initial struggle to attune to speech sounds, related to a more complex acoustic environment. Notably, immature neural responses to phonemic contrasts during the early months of life do not necessarily predict poorer language outcomes. Instead, they seem to facilitate latter attunement, leading to faster maturation rates and stronger responses later in development that are associated with enhanced language outcomes (García-Sierra et al., 2021; Schaadt et al., 2015, 2023; Werwach et al., 2022). In contrast, monolingual infants in our sample followed an inverted U-shaped trajectory for F_0_ encoding, with spectral amplitudes increasing from birth to 6 months and then declining between 6 and 12 months. These opposite trajectories in F_0_ encoding depending on bilingual experience parallel those depicted for phonetic contrast discrimination during the second half of the first infant year (Byers-Heinlein & Fennell, 2014). Our findings support and extend prior evidence indicating that the sensitivity to the lexical nativeness of speech sounds emerges at around 6 months of age (Cheng et al., 2013; Novitskiy et al., 2022). F_0_ variability may serve as a salient perceptual attribute that facilitates language separation for bilingual infants (Liu & Kager, 2017).

Bilingual experience further affected the maturation of formant structure encoding, with distinct effects on each vowel's F_1_ frequencies. Specifically, infants with over 7 months of total bilingual exposure showed linear growth in spectral amplitudes for /o/ F_1_ across the first year, whereas this linear effect of age was not significant for monolingual infants. In contrast, neural encoding of /a/ F_1_ followed a linear trajectory regardless of the amount of bilingual exposure. Our findings suggest a stronger influence of bilingual exposure in lower versus higher frequencies during early infant ages that may be related to the previously mentioned spectrally ascendant pattern of development (Graven & Browne, 2008). However, although describing distinct developmental trajectories for the encoding of pitch and formant structure, our results demonstrate that bilingual exposure positively impacts the neural encoding of both speech features at the age of 12 months. We thus provide evidence for previous literature hypothesizing a heightened acoustic sensitivity in bilingual infants manifesting near the end of the perceptual reorganization process (Liu & Kager, 2015, 2017), by specifically signaling distinct neural attunement along the auditory pathway associated with early bilingual exposure. Additionally, our results complement previous research on the effects of prenatal bilingual exposure in neural encoding of speech sounds (Gorina-Careta et al., 2024) by extending the scope to postnatal development throughout the first year of life.

Our findings further underscore a significant influence of sex on the neural speech encoding in infants. Female infants exhibited higher overall spectral amplitudes at the stimulus F_0_, indicating superior voice pitch neural phase-locking throughout the first year compared to males. Additionally, females followed a distinct quadratic developmental trajectory for pitch encoding, surpassing male spectral amplitudes at F_0_ by 6 months and then declining closer to male values by 12 months. A similar quadratic pattern was observed for females in the encoding of /o/ F_1_, marked by significant improvements during the first 6 months of life. Female infants also displayed overall higher amplitudes at /a/ F_1_ across the first year. These results extend those of Krizman et al. (2019, 2020) by revealing sex differences in auditory processing emerging in infancy, well before the adolescent differences they documented.

These sex-specific developmental patterns highlight early differences in neural encoding of speech-acoustic features between male and female infants during the first year of life. Our results align with previous research reporting sex differences in early speech encoding and acquisition, including disparities in the maturation of involved brain areas (Alexopoulos et al., 2022), infant phonological discrimination (Friederici et al., 2008), the onset and rate of word production (Dailey & Bergelson, 2023), and the frequency of speech-like vocalizations before first words (Sung et al., 2013). A growing body of research links infants' hormone levels to these early developmental differences (Lust et al., 2010; Lutchmaya et al., 2001; Wermke et al., 2014). For example, sex hormone levels have been linked to infant phonological discrimination (Friederici et al., 2008), articulatory skills (Quast et al., 2016), and later language abilities in childhood (Hollier et al., 2013; Schaadt et al., 2015).

Another factor potentially contributing to these differences is related to caregivers’ speech input. Studies suggest that caregivers use higher pitch and a greater pitch range when speaking to female infants compared to males, with this difference growing over time (Kitamura & Burnham, 2003; Kitamura et al., 2001). The duration of speech input also shows sex-specific patterns, with speech directed to male infants decreasing during the first year of life, while increasing for female infants (Sung et al., 2013). Furthermore, caregivers tend to talk more to infants who have started talking (Dailey & Bergelson, 2023), which could reinforce sex differences in speech abilities given that females often begin speaking earlier than males (Bornstein et al., 2004; Eriksson et al., 2012; Fenson et al., 1994). These biological and social factors likely interact, shaping the sex-related differences in the developmental trajectory of neural speech encoding observed in our results.

Although there is a large research community exploring speech perception in infants, using both behavioral and neurophysiological measures (Byers-Heinlein et al., 2010; Hervé et al., 2022; Kuhl et al., 2003, 2006; Kujala et al., 2023), the use of the FFR provides a direct neural measurement of the auditory hierarchy that reflects the attunement to the spectral and temporal features of speech (Coffey et al., 2016; Gorina-Careta et al., 2021; Krizman & Kraus, 2019; Skoe & Kraus, 2010). By utilizing the FFR, our results highlight the pivotal importance of the first 6 months in neural development for speech encoding. Thereby, future studies should incorporate more frequent time-point measurements to gain a comprehensive understanding of this early period.

### Future Directions

We observed distinct developmental trajectories in the neural encoding of speech features, influenced by both sex and perinatal bilingual exposure. Nonetheless, these effects may not be entirely independent from other experiential or socioeconomical factors, such as cultural practices, parental education, or family income. While these variables were not explicitly controlled in the current design, future research should address them to more precisely isolate the contribution of bilingual exposure and sex. Consistently incorporating these factors into research on early speech and language development is crucial for capturing an accurate depiction of these early processes, enhancing replicability and deepening our understanding of infant development.

In our results, bilingual infants exhibited reduced pitch encoding at 6 months, a difference that reversed by 12 months, suggesting a slower but possibly more refined developmental trajectory. However, our study did not directly account for the acoustic content of the languages to which infants were exposed. Bilingual contexts create a complex acoustic environment, and determining which particularities drive these developmental shifts is essential. For instance, do these effects arise from greater exposure to shared features across languages, from the richness and diversity of acoustic cues provided by two linguistic systems, or from the relative balance of input across languages? Disentangling these possibilities will aid at clarifying which elements of bilingual, and even multilingual, contexts shape early neural encoding of speech.

Similarly, understanding the mechanisms through which sex influences neural speech encoding is critical. In our results, female infants showed generally stronger neural encoding during the first year of life, with a marked peak at 6 months that converged with males by 12 months. To better understand these distinct developmental dynamics, future studies incorporating hormonal measures across multiple time points and employing complementary neuroimaging techniques could provide valuable insight into the underlying functional and structural mechanisms driving these sex-related patterns.

Ultimately, identifying how early neural encoding of speech is shaped by sex and linguistic environment could help tailor screening and support practices to better accommodate individual variability during the first year of life. These insights could have meaningful implications for at-risk populations where speech acquisition may be delayed or atypical.

### Conclusion

The present study provides novel insights into the distinct developmental trajectories of neural speech encoding during the first year of life, influenced by both sex and perinatal bilingual exposure. By examining longitudinally FFR neural responses to a two-vowel stimulus, we revealed a significant maturation of pitch and formant structure encoding throughout the first 6 months of life, without further maturation up to the age of 12 months. These findings emphasize the first 6 months as a sensitive period for neural adaptation to the acoustic environment, contributing to early phonetic learning and language acquisition. The moderating effects of bilingual exposure on voice pitch and formant encoding, as well as the sex-specific differences observed, underscore the complexity and individual variability related to early auditory and speech perception development. These results not only extend previous research but also contribute to a deeper understanding of the neural foundations of early language acquisition. Future studies incorporating more frequent developmental assessments could help refine our understanding of this sensitive period.

## ACKNOWLEDGMENTS

The authors want to express their sincere gratitude to the many families who generously participated in this research project and contributed at any stage of the longitudinal follow up.

## FUNDING INFORMATION

Carles Escera, Ministerio de Ciencia, Innovación y Universidades (https://dx.doi.org/10.13039/100014440), Award ID: projects PGC2018-094765-B-I00 [MCIN/AEI/10.13039/501100011033/FEDER “Una manera de hacer Europa”] and PID2021-122255NB-100 [MCIN/AEI/10.13039/501100011033/FEDER, UE]. Carles Escera, María de Maeztu Center of Excellence, Award ID: CEX2021-001159-M by MCIN/AEI/10.13039/501100011033. Carles Escera, Consolidated Research Group of the Catalan Government.

## AUTHOR CONTRIBUTIONS

**Marta Puertollano**: Conceptualization: Equal; Data curation: Lead; Formal analysis: Lead; Investigation: Equal; Methodology: Equal; Visualization: Lead; Writing – original draft: Lead. **Natàlia Gorina-Careta**: Conceptualization: Equal; Data curation: Equal; Investigation: Equal; Methodology: Equal; Project administration: Equal; Supervision: Equal; Writing – review & editing: Equal. **Siham Ijjou-Kadiri**: Conceptualization: Equal; Data curation: Equal; Investigation: Equal; Methodology: Equal; Visualization: Supporting; Writing – review & editing: Supporting. **Alejandro Mondéjar-Segovia**: Data curation: Equal; Investigation: Equal; Methodology: Equal; Writing – review & editing: Supporting. **María Dolores Gómez-Roig**: Funding acquisition: Supporting; Investigation: Supporting; Project administration: Equal; Resources: Lead; Supervision: Equal; Writing – review & editing: Supporting. **Carles Escera**: Conceptualization: Lead; Data curation: Equal; Formal analysis: Equal; Funding acquisition: Lead; Investigation: Equal; Methodology: Lead; Visualization: Equal; Writing – review & editing: Lead.

## DATA AVAILABILITY STATEMENT

The data and the analysis code that support the findings of this study are available on OSF (https://osf.io/uxd7b/).

## TECHNICAL TERMS

Frequency-following response (FFR): A neural signal that mirrors the frequency and timing features of sound, reflecting neural-level auditory processing.
Formant: A concentration of acoustic energy at specific frequencies that defines the distinct qualities of vowel sounds.
Pitch tracking: The neural encoding of the changes in the fundamental frequency of a sound over time.
Neural phase-locking: The precise timing of neural firing to the periodic elements of a sound wave.
